# The Impact of Intimate Partner Violence on Young Women’s Educational Well-Being: A Systematic Review of Literature

**DOI:** 10.1177/15248380211052244

**Published:** 2021-12-11

**Authors:** Lucia E. Klencakova, Maria Pentaraki, Cathal McManus

**Affiliations:** 1 School of Social Sciences, Education and Social Work, 1596 Queen’s University, Belfast, United Kindgom

**Keywords:** IPV, intimate partner violence, domestic violence, young women, education impact

## Abstract

Research highlights that romantic relationships of young people are not all ‘puppy love’ but can be also abusive. Intimate partner violence (IPV) is a gendered phenomenon as it primarily affects women who are at a higher risk of more severe forms of violence and also suffer more severe consequences than young men. IPV leads to substantial negative outcomes such as mental health decline, economic insecurity and/or academic underachievement. Particularly for young females, education is a powerful protective factor against re-victimisation and economic dependence which often forces women to remain trapped in abusive relationships. This review was conducted to integrate and summarise research available on IPV and its impact on young women’s educational well-being to fill a significant gap in the literature. Under the guidance of PRISMA, terms related to the criteria of young women aged 10–24, IPV and education were searched in the databases EBSCO, PsycINFO, Scopus, ProQuest and CINAHL. While the initial search yielded 6005 articles, we were left with only 10 articles for the analysis. In summary, the evidence suggests that females tend to display issues around concentration, absenteeism and academic disengagement, as well as decline in performance such as failing grades and higher drop out rates.

## Literature Review

During adolescence, young people develop their first romantic relationships. These romantic relationships contribute to their relational development and can be a defining characteristic of young people’s lives (Connolly et al., 2014). Research highlights that the romantic relationships of young people are not all ‘puppy love’ but can be also abusive (Arqimandriti et al., 2018; Barter et al., 2009). Abuse in young people’s romantic relationships is part of the social problem widely described as domestic and intimate partner violence (WHO, 2017), frequently used interchangeably to refer to ‘behaviour within a [romantic] intimate relationship that causes physical, sexual or psychological harm, including acts of physical aggression, sexual coercion, psychological abuse and controlling behaviours’ (World Health Organization, 2010).

This abuse can be experienced in short- and long-term, casual and serious relationships, both online and face-to-face. To better understand intimate abuse among young people, some studies explored when IPV was most active and found that almost 65% of first victimisation happened between the ages of 13–19 with peak ages of perpetration ranging from 16 to 17 years (Arqimandriti et al., 2018; Bonomi et al., 2012). Research highlights the extent to which IPV is a significant issue among young people (Duval et al., 2018; Korkmaz, 2017) with IPV prevalence rates reported at 20–26% in Australia (Dillon et al., 2015), 42.9% in Norway (Hellevik & Överlien, 2016), and 45% in the UK and the US (Fox et al., 2014; Gehring & Vaske, 2017).

IPV is a gendered phenomenon as it primarily affects women and to a lesser degree, men. National Crime Surveys, such as the British Crime Survey, consistently highlight that women are the majority of survivors of abuse (Home Office, 2016; ONS, 2018). Globally, one in three women experience sexual and/or physical violence by an intimate partner during her lifetime (WHO, 2017). Similarly, IPV in young people’s relationships has gendered characteristics. Research suggests that young women are at a higher risk of more severe forms of IPV than young men (Boney-McCoy & Filkenhor, 1995; Diette et al., 2017) and suffer more severe consequences than men (Barter et al., 2009; Jackson et al., 2000; Reyes et al., 2018; Stermac et al., 2018).

### The Impact of IPV on Young Women

Research has not yet come up with a universally accepted definition nor a conceptual framework that would encompass the complexity of IPV (Burelomova et al., 2018). To date, the most common conceptual definition used to measure IPV is physical violence, including sexual (Duval et al., 2018; Postmus et al., 2020; Stermac et al., 2018). There is a consensus that physical and sexual violence lead to mental health decline, substance abuse and behavioural issues (Garnefski & Arrends, 1998; Jordan et al., 2014); however, the invisible forms of abuse, including coercive control, also severely disadvantage females long-term (Collins et al., 2009; Postmus et al., 2020; Voth Schrag & Edmonton, 2017).

More specifically, the hidden forms of IPV such as deliberate neglect of child rearing duties, forced pregnancy, and/or economic abuse and academic sabotage lead to substantial negative outcomes such as mental health decline, economic insecurity and/or academic underachievement (Banyard & Demers, 2017; Voth Schrag & Edmonton, 2017; Postmus et al., 2020). Several studies suggested that young women who experienced IPV during early years were more likely to be subjected to subsequent abuse in adulthood (Arqimandriti et al., 2018; Capaldi et al., 2012; Duval et al., 2018). Few studies also concluded that a lower educational level increased the likelihood of IPV (re-)victimisation (Bonnes, 2016; Kreager et al., 2013). In addition to experiencing a vicious cycle of IPV, financial dependency often forces women to remain trapped in abusive relationships (Brush, 2004; Postmus et al., 2020).

### IPV and Education

Education has been discussed as one of the most powerful protective factors against IPV (Dank et al., 2014; Duval et al., 2018) and the strongest empowerment tool in overcoming domestic violence trauma (Cattaneo & Goodman, 2015; Jain & Singh, 2016). The repercussions of IPV identified above provide some indication on how they might potentially impact upon the education of young women. According to Sterne and Poole (2010), some pupils will treat education as an escape, a safe place, and will overcommit to schoolwork and life, while the majority will exhibit rage and aggression accompanied by scholastic difficulties, becoming withdrawn and disengaged. Some studies highlight additional outcomes among young women such as a lower GPA, truancy or college drop out which often lead to lower income and/or poverty later in life (Brush, 2004; Jordan et al., 2014; Hicks, 2006).

Although a few systematic reviews examined the myriad forms of violence in childhood and adulthood and their impact on victims (see, for instance, Alhabib et al., 2010; Duval et al., 2018; Fry et al., 2018), the correlation between IPV experiences and their impact on women’s educational attainment remains comparatively unexplored (Collins et al., 2009; Voth Schrag & Edmonton, 2017). A number of studies examined IPV among young people and its impact on their educational attainment’; however, other types of abuse such as bullying and/or assault by a peer were added into the measurement (Hammig & Jozkowski, 2013; Stermac et al., 2018; Umana et al., 2014). Consequently, educational outcomes such as poor grades, leaving school or an inability to concentrate or complete work cannot be attributed to the IPV experience with absolute certainty.

Some studies examined IPV victimisation and its impact on education merged with economic prosperity (Fernandez et al., 2015; Macmillan and Hagan, 2004). Researchers have concluded that experiencing IPV negatively impacts educational performance which has serious long-term implications such as financial difficulties or diminishing employment opportunities in adulthood with the subsequent financial repercussions this generates (ibid). Other studies focused on IPV and its impact on educational attainment; however, researchers incorporated a sample of women that spanned from adolescents to pensioners (Brush, 2004; Hicks, 2006). Therefore, this review was conducted to synthesise available literature to increase our understanding of the impact of IPV on young females’ educational well-being and attainment.

## Methodology

This review aims to fill a significant gap in the literature by examining young females’ experiences of IPV and its impact on their educational attainment. We systematically identify and summarise existing research to (a) gather evidence of the educational outcomes after experiencing IPV among young women and (b) to draw conclusions and propose recommendations about research gaps and limitations, all whilst minimising selection bias. As a result of the above, two research questions were posed prior to the literature search: RQ1: *What are the consequences of IPV on young women’s education?* RQ2: *What conclusions can be drawn from this literature review: (i) literature gaps and future research, and (ii) limitations?*

### Search Strategies

A systematic search was conducted in November 2018, employing the specifications outlined by PRISMA, or the Preferred Reporting Items for Systematic Reviews and Meta-Analyses (Liberati et al., 2009). The search was performed in the following electronic databases: EBSCO, PsycINFO, Scopus, ProQuest and CINAHL.

### Search terms included were:

• ‘Intimate partner violence’ or ‘dating violence’ or ‘domestic violence’ or ‘relationship violence’ or ‘interpersonal violence’
• Women or woman or girl* or female*
• Teen* or adolesc* or youth or ‘young people’ or young
• University or school or educat* or academ* or college or postsecondary

In line with previous research, searched terms *dating* or *interpersonal violence* and *relationship violence* or *abuse* are commonly used in the USA (Korkmaz, 2017; Meiksin et al., 2020); however, in some languages or cultures ‘dating’ has no equivalent or is expressed and understood differently; hence, the more widely used terms in Europe and internationally *intimate partner violence* (IPV) or *intimate abuse* were also included (Barter, 2009; Korkmaz, 2017). The term domestic violence, while typically associated with adult samples, also often includes adolescents aged 16 years and over (Alhabib et al., 2010; Messinger et al., 2014). Following the database search, a hand search was conducted in reference lists of all articles selected for final analysis as well as citation indexes, Google Scholar and Scopus.

### Eligibility Criteria

Eligible studies were included if they were published in English in a peer-reviewed journal in or after 1981 until 2018. Due to the fact that the first known English academic research publication acknowledging IPV in young people’s romantic relationships was published in 1981, this year was chosen as the starting point (Makepeace, 1981). As the literature has highlighted, females are likely to experience more negative outcomes as a result of IPV and hence, the search included research articles with samples or easily identified subsample of girls and young women. As there are 11% of girls in African regions and 5% of children globally married before the age of 15 (UNICEF, 2020) and child brides as young as 10 years old (DeGroot et al., 2018; Nour, 2009), the age range was set between 10–24 as it pertains to the World Health Organization’s definition of young people.

Eligible studies must have been related to intimate partner violence and must have simultaneously explored either educational, academic or school-related outcomes, consequences and/or sabotage. Studies were included if they defined IPV as violence or abuse between a young female and an intimate partner; domestic violence within a parental home or bullying and such was not included. Studies were also excluded if their sole focus was on male participants, if they were published before 1981 or included a sample outside the 10–24 years of age range (unless participants were reflecting retrospectively on their experiences within the pre-established age range). Studies that did not address intimate partner violence among young people or within their relationships as well as its impact on education were also excluded.

### Selection of Studies and Data Extraction

The initial search yielded 6005 articles. After removing duplicates, we were left with 4003 studies. Reviewing titles resulted in 289 abstracts that were screened by two independent reviewers. Excluded titles related to healthcare sector such as sexual assault and HIV, also family domestic violence, attitudes towards domestic violence/IPV, help seeking and disclosure, risk and protective factors, typologies of perpetrators or violence, and/or IPV prevalence. Excluded abstracts focused on perpetrators, incident and re-victimisation, and different types of violence such as sexual assault, rape or harassment but not violence by an intimate partner, or did not mention school and/or academic or educational impact or consequences. The final number of articles meeting the criteria was 22.

**Figure 1.**
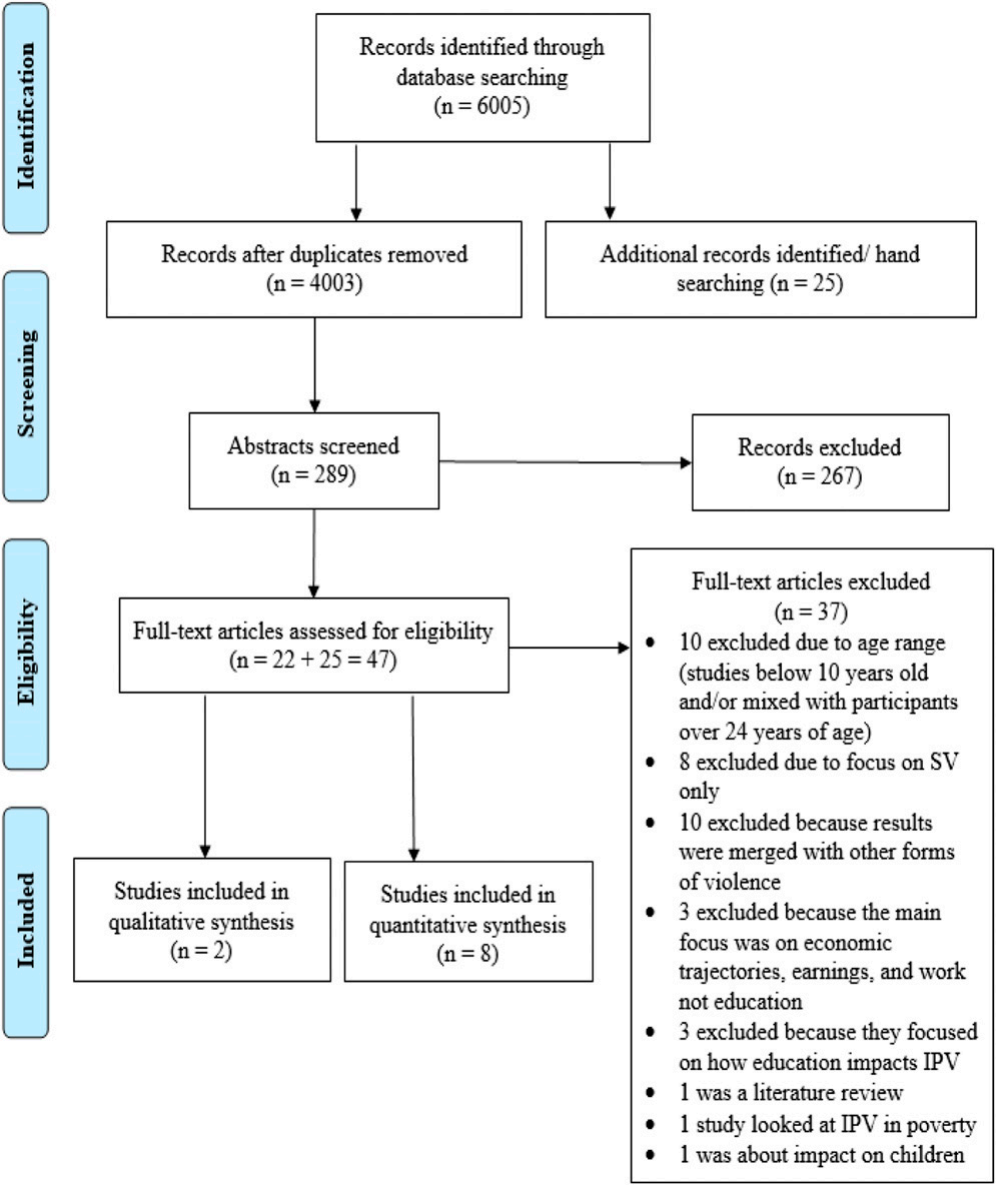
PRISMA flow chart.

Twenty-five articles emerged from secondary searches in citation indexes and reference lists of all articles selected for a full review; this left a total of 47 articles for a final full-text review (*n* = 47). Each reviewer screened the articles to determine eligibility for further analysis; out of these, 10 were excluded due to a sample mixed with participants outside the pre-established age range, and 18 because IPV was merged with different types of violence or the focus was solely on domestic or sexual violence (including rape and harassment by a peer or stranger); articles that focused on outcomes other than scholastic or educational, such as the impact of IPV on finances, earnings, children or poverty, were also excluded (*n* = 5). Further, reviewers agreed on eight articles, while six were discussed until a consensus was reached which left a total of 10 articles for the analysis and critical appraisal (see Figure 1: PRISMA Flow Chart).

Critical appraisal was conducted by 2 reviewers; a quality assessment for qualitative studies was guided by the checklist developed by the Critical Appraisal Skills Programme (CASP, 2018) and Quality Assessment Tool for Observational Cohort and Cross-Sectional Studies was used to guide an assessment of quantitative and longitudinal studies (National Institute of Health, n.d.). Of the final 10 articles, none were deemed poor; both reviewers scored 70% of articles as good and 30% scored as fair. All articles were deemed of satisfactory quality to be included in the final analysis.

## Results

### Studies’ Overview

Of the 10 studies included in the systematic review, eight were quantitative and two, qualitative. Three studies were longitudinal (Adams et al., 2013; Foshee et al., 2013; Strenio, 2017), the rest of the studies were cross-sectional (Banyard & Cross, 2008; Brewer et al., 2018; Chronister et al., 2014; Dank et al., 2014; Edwards, 2018; Martz et al., 2016; Wiklund et al., 2010). Nine studies were performed in the USA and one in Sweden (Wiklund et al., 2010). Seven of the studies were published between 2013 and 2018, with the first two relevant studies published in 2008 and 2010 (Banyard & Cross, 2008; Wiklund et al., 2010). It is worth noting that eight articles had a significant gap between the initiation of the study and the year of publication; on average, this gap was 5–8 years with Foshee et al. (2013) and Adams et al. (2013) initiating data collection 10+ years prior to the publication.

### Sample Characteristics

The sample context was rather congruent with seven studies focusing on secondary education, middle and high schools (Banyard & Cross, 2008; Dank et al., 2014; Edwards, 2018; Foshee et al., 2013; Martz et al., 2016; Strenio, 2017), including recruitment of high school students at secondary education establishments and youth clubs (Chroniser et al., 2014); one study focused on college students (Brewer et al., 2018) and one study was conducted at a youth health centre (Wiklund et al., 2010). Further, settings explored were urban and suburban (Adams et al., 2013; Banyard & Cross, 2008; Brewer et al., 2018; Wiklund et al., 2010), rural areas (Foshee et al., 2013; Martz et al., 2016) and three studies included both urban and rural settings (Chronister et al., 2014; Dank et al., 2014; Strenio, 2017); one study did not specify but it appears all types of settings were included (Edwards, 2018).

In total, 124,956 participants aged 11–24 were included across nine studies (Adams and colleagues recruited women aged 18–54 who recounted their experiences of adolescent IPV); three studies recruited a female-only sample, whilst the other seven studies included both young female and male participants – the ratio of participants was approximately the same in six out of seven remaining studies (see Table 1 for sample overview). With the exception of Banyard and Cross (2008) and Strenio (2017), all studies obtained data on participants’ race and ethnicity; three studies collected information about sexual orientation with over 90% of participants in all studies self-categorising as heterosexual (Brewer et al., 2018; Dank et al., 2014; Edwards, 2018). In summary, samples were homogenous, largely lacking in representation of sexual, racial and ethnic minorities.

**Table 1.** Demographic information of participants.

	Adams	Banyard	Brewer	Chronister	Dank	Edwards	Foshee	Martz	Srenio	Wiklund
Particpants (n)	498	2101	85,071	19	3745	25,122	3328	1003	4565	2
Female	100%	51%	67.2%	100%	52.3%	50.8%	51%	52%	55.4%	100%
Transgender					0.5%					
Heterosexual			90%		93.8%	90.5%				
LGBTQ			10%		6.2%	9.5%				
Caucasian			65.2%	13 (70%)	73.7%	88%	43%	92.8%		*Swedish
Afr. American	56%		23.9%		5%	2.2%	50%			
Latino/a				3(16%)	8.2%					
Asian				1	2.2%	1.9%				
Native American				1	0.7%	1.4%				
Other			11% (multiracial)	1 (Jewish)	10.2% (multiracial)	7% (Latin, Asian, and mixed race)	7.2% (non-white)			

Dank et al. (2014) was the only study that included transgender as a category, and significantly, it concluded that transgender youth were the most likely group to both perpetrate and be subjected to victimisation in three of four forms of abuse (prevalence of victimisation among women and transgender youth was, respectively, at 23.9% and 88.9% for physical, 16.4% and 61.1% sexual, 49.7% and 58.8% psychological and 28.8% and 56.3% for cyber abuse; perpetration: physical 25.5% vs. 58.8%, sexual 1.2% vs. 17.6% and cyber 13.9% vs. 35.3%). While female participants in the study reported perpetrating psychological abuse at a slightly higher rate than male or transgender participants (31.7% females vs. 29.4% transgender), there is a lack of clarity surrounding motivations for perpetrating and/or whether couples were heterosexual or same-sex. Indeed, most articles lack contextual and situational factors that would explain the experience more precisely; this will be further explored in the Discussion section.

### Summary of Constructs and Measures (*n* = 10)

Two distinct constructs emerged during the analysis: Six out of 10 studies used dating violence or abuse to designate IPV among young people; although Chronister et al. (2014) labelled IPV as unhealthy relationships to encourage a multiplicity of abuse experiences, authors refer to the phenomenon as dating violence. Three studies defined the experience as intimate partner violence or abuse (Adams et al., 2013; Brewer et al., 2018; Strenio, 2017), while the European study referred to IPV as interpersonal violence (Wiklund et al., 2010). Essentially, the literature would appear to show that the preferred term for IPV in the US is *dating violence* or *abuse (victimisation).* The studies highlighted a diversity of views on how IPV manifested itself. In four out of 10 studies, abuse was measured as physical and sexual violence (Banyard & Cross, 2008; Edwards, 2018; Martz et al., 2016; Strenio, 2017), whilst five studies added psychological abuse (Adams et al., 2013; Brewer et al., 2018; Chronister et al., 2014; Dank et al., 2014; Foshee et al., 2013). Dank et al. (2014) also included cyber abuse.

Four studies that measured IPV as physical and sexual violence, analysed a subsample of 2101 participants (of the 9791 original sample) who had no missing data on dating history and reported 16.9% physical and 13.2% sexual victimisation among 516 participants (Banyard & Cross, 2008); in Edwards’ (2018) study, 64% of the original 38,181 surveys led to reporting prevalence of physical and sexual violence at 9.1% and 14.1% (heterosexual), 25.3% and 28.2% (bisexual), 20.2% and 26.5% (questioning) and 21.9% and 16.6% (lesbian); out of 78.2% (*n* = 1003), 12.1% of participants reported being abused physically and 11.3% sexually, with 4% having experienced both types of violence (Martz et al., 2016); and 34% of the original sample of women (*n* = 4565) indicated experiencing both physical and sexual abuse in Strenio (2017). Wiklund et al. (2010) interviewed participants who experienced gender-related subordination, describing their former relationships as controlling or violent (guarded, beaten, sexually degraded and ‘everything except killing’).

In Adams et al. (2013), participants retrospectively reflected on their IPV experiences during adolescence; the response rate was 86 – 93% over five waves with a total of 498 participants who experienced IPV, which accounted for 16% of the original sample of participating women. The longitudinal study by Foshee et al. (2013) recorded the average prevalence of physical (including sexual) and psychological IPV at 19.5% and 32.5% among eighth graders, 20.5% and 35.5% among ninth graders, 20.5% and 42% among 10th graders, 20% and 46% among 11th graders and 18% and 43% for 12th grade students. Brewer et al.’s (2018) analysis of 85,071 young people, including 67.2% of females, revealed 2% of sexual violence (forced into sex or unwanted acts), physical abuse (2.1%) which included hitting, kicking or slapping and emotional abuse at 9% (a dichotomous yes–no response question, ‘were you a victim of emotional aggression and stalking?’).

### Education Measures

In relation to education, four studies focused specifically on academic performance (Brewer et al., 2018; Dank et al., 2014; Edwards, 2018; Martz et al., 2016), while three studies (Banyard & Cross, 2008; Chronister et al., 2014; Foshee et al., 2013) identified targeted outcomes and defined them as academic consequences or impact. The studies that defined education in purely performance-related terms, measured the impact with grades awarded; Dank et al. (2014) added attendance, and Brewer et al. (2018) explored difficulties at school. The other studies focusing on consequences measured the impact as grades (Banyard & Cross, 2008; Chronister et al., 2014; Foshee et al., 2013), school attachment (Banyard & Cross, 2008), attendance (Chronister et al., 2014), aspirations (Chronister et al., 2014; Foshee et al., 2013; also Strenio, 2017) and ‘performance’ as the ability to concentrate or complete homework (Chronister et al., 2014).

**Figure 2.**
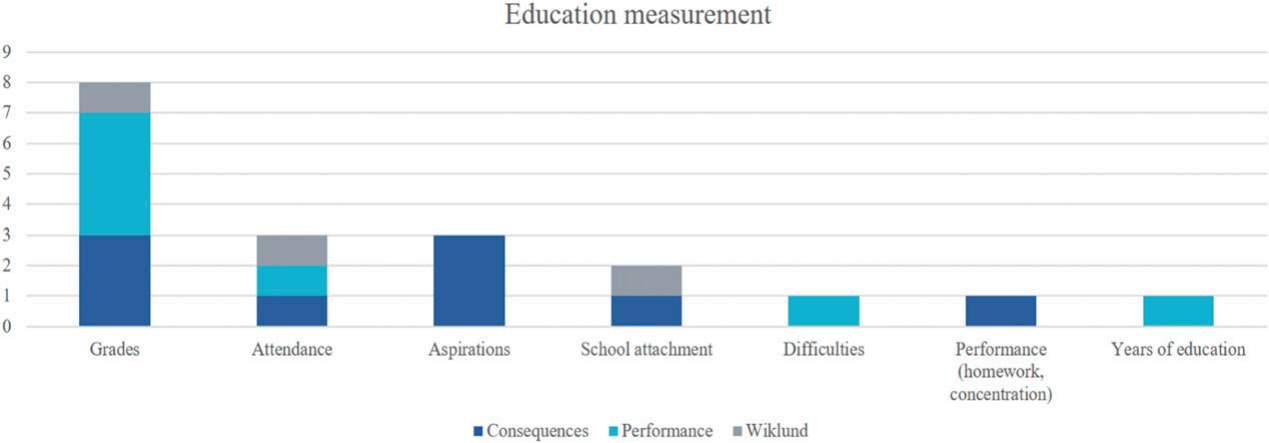
Education measurement.

Educational attainment was captured as the highest number of years of education completed in the study by Adams and colleagues (2013), while Wiklund et al. (2010) concluded through thematic analysis that attendance and school attachment declined in both participants after experiencing IPV, which ultimately impacted women’s grades due to skipping school or eventually dropping out. Brewer et al. (2018) examined the issue of difficulties at school with simple yes and no answers to ‘… have academics been very difficult for you to handle?’ which leaves obvious questions around what was deemed to constitute ‘difficulty’ in the eyes of respondents (see Figure 2: Education measurement).

### Education-Specific Outcomes

The repercussions of IPV identified and collated in the chosen studies highlight that students tend to demonstrate concentration issues, absenteeism, academic disengagement, decrease in grades and higher dropout rates (Banyard & Cross, 2008; Brewer et al., 2018; Wiklund et al., 2010). Seven out of 10 studies reported falling grades (except Foshee et al., 2013) and concluded that physical violence had a direct, negative impact on young women’s GPA; some attributed a decline in academic attachment, including attendance, as well as school difficulties to physical violence such as abusers’ manipulation, sabotage or physical restraint (Banyard & Cross, 2008; Chronister et al., 2014; Dank et al., 2014; Wiklund et al., 2010). Both Strenio (2017) and Adams et al. (2013) concluded that IPV experiences significantly diminish educational attainment (Table 2 includes a summary of education outcomes by abuse type).

**Table 2.** Type of abuse and its outcome(s).

Type of abuse	Outcomes	Authors
Physical	Declines in grades or GPA	Banyard, Brewer, Chronister, Dank, Edwards, Martz, and Wiklund
	Thoughts of/dropping out	Banyard and Wiklund
	Lower academic aspirations	Chronister, Martz, and Strenio
	Lower school attachment	Banyard, Chronister, and Wiklund
	Academic difficulties	Brewer
	Lower attendance/truancy	Chronister, Dank, and Wiklund
	Lower educational attainment (education completion)	Adams and Strenio
Sexual	Decline in graded or GPA	Banyard, Brewer, Chronister, Edwards, Martz, and Wiklund
	Thoughts of/dropping out	Banyard and Wiklund
	Lower academic aspirations	Banyard, Chronister, and Wiklund
	Academic difficulties	Brewer
	Lower attendance/truancy	Chronister
	Lower educational attainment (educational completion)	Adams and Strenio
Psychological	Decline in grades or GPA	Brewer, Chronister, and Wiklund
	Lower academic aspirations/drop out	Chronister and Wiklund
	Lower school attachment	Chronister and Wiklund
	Academic difficulties	Brewer
	Lower attendance/truancy	Chronister, Dank, and Wiklund
	Lower educational attainment (education completion)	Adams
Cyber	Declines in grades or GPA	Dank
	Low attendance/truancy	Dank

Further, the majority of studies linked truancy and/or drop out to physical force or restraint and/or psychological abuse such as being forced to use substances and/or to participate in delinquent or illegal activities (Banyard & Cross, 2008; Chronister et al., 2014; Dank et al., 2014; Wiklund et al., 2010). These findings were expanded upon in Banyard and Cross (2008) and Chronister et al. (2014), which found that low school attachment and negative perceptions of the school environment stemming from IPV experiences, often resulted in poor grades and drop out. Low attendance and attachment as well as decline in grades were especially significant for females belonging to sexual and racial minorities; for example, Dank et al. (2014) concluded 10 times higher percentage of failing grades (Ds/Fs at 10.6%) among LGBTQ as opposed to heterosexual participants (1.5%).

Additionally, in Chronister et al. (2014), females struggled to keep up with schoolwork and experienced concentration issues and low motivation to continue studying and pursue future endeavours as a result of the experience; particularly, psychological abuse was predominantly causing women to perceive themselves as ‘only good for stripping’ or being bullied into believing that they are ‘never going to be anything’ (Chronister et al., 2014; Wiklund et al., 2010). Similarly, sexual violence and coercion led to the same outcomes; for instance, a history of rape and/or IPV predicted a lower probability of attending college for young females, and was also associated with lower grades in school – females indicated a decline from A- or B-level to C-level and below with a significant relative ratio 1.07–1.15, which was not statistically significant for males (Martz et al., 2016).

According to Strenio (2017), IPV is significantly associated with a decreased likelihood of graduating high school (reduced probability by 5.64 percentage points) and reduces the probability of college graduation (8.53 percentage points). Increase in violence intensity (severity and chronicity) was statistically significant in reducing the likelihood of education completion (ibid). Although women completed, on average, 6–16 years of education in a study by Adams and colleagues (2013), the researchers concluded that adolescent IPV was negatively correlated with educational attainment in that women victimised during adolescence obtained 0.5 fewer years of education than women with no IPV experience. The authors also indicated that the implication of lower educational attainment leads to a long-term economic disadvantage, stating that ‘IPV survivors earned significantly less and experienced significantly less growth in earnings over time as a consequence of lower educational attainment’ (p. 3295).

Only one study measured the impact of cyber abuse on academic performance and concluded that abusers’ threats and online harassment negatively affected attendance and therefore grades (Dank et al., 2014). Brewer and colleagues (2018) tested whether poor physical health influenced academic performance and found a minimal impact as opposed to IPV which caused the study’s participants to report a low GPA and difficulties at school, including managing school workload (Brewer et al., 2018).

Five studies further highlighted a decrease in educational performance (Brewer et al., 2018; Chroniser et al., 2014; Dank et al., 2014; Edwards, 2018; Martz et al., 2016). However, there are a number of important differences between definitions preferred by each study; for instance, while all studies measured performance as grades or GPA, attendance and academic difficulties were added as an additional measure (Brewer et al., 2018; Dank et al., 2014). Chronister and colleagues also added drop out or expulsion, future aspirations and concentration, including homework completion, while two studies that indicated a decline in educational performance measured this as grades only, without considering the wider context of scholastic aspirations, such as motivation and engagement, concentration or attendance (Edwards, 2018; Martz et al., 2016).

Fundamentally, young females in all studies suffered negative educational consequences with only one study, Foshee et al.’s (2013) longitudinal analysis, suggesting that there was no significant impact on educational outcomes. The study asked young people to indicate how important it is to graduate from high school and go to college, coded as important and not important (0 and 1, respectively) as well as self-report letters in four subjects to obtain an approximate GPA. The authors concluded that neither psychological nor physical violence, including sexual, predicted low academic grades or decline in academic aspirations over the longer term. However, the study inferred that psychological abuse leads to detrimental consequences in the form of substance abuse and mental illnesses (depression/anxiety), especially significant for female participants, which may impact academic outcomes negatively. A summary of the key findings can be found in Table 3.

**Table 3.** Summary of key findings.

Author(s)	Sample	Study characteristics	Types of violence studied	Educational outcomes explored	Summary of critical findings		
					generic consequences	educational consequences	limitations and future research
Adams et al. (2013)	Analysed sample: 498 single mothers who received cash assistance from Temporary Assistance to Needy Families (welfare) Age: 18–54 (stratified random sampling) Gender: Females only Ethnicity: 56% African-American Sexual orientation: N/A	Study design: Quantitative, longitudinal, survey Setting: urban and suburban; Women’s Employment Study in Michigan, USA Years of data collection: 1997–2002	Adolescent IPV: Physical Sexual IPV Psychological abuse Measured with a binary variable ‘yes, no’, defining the experience as beating, pushing, sexually forcing, threats or harm	Correlation between IPV, educational attainment and earnings: The highest number of years of education completed	Education significantly related to annual earnings and growth in earnings over time: over the course of 4-year study, adolescent IPV victims earned less $343 and $442 less growth in earnings	Diminished educational attainment (women victimised during adolescence obtained 0.5 fewer years of education)	Limitations: Time lag (data collection 1997 but published in 2013), low generalisability and a lack of covariates (family and health) Future research: Diverse sample, more focus on education and long-term impacts, and more outcome measures
Banyard and Cross (2008)	Analysed sample: 2101 convenience subsample of Teen Assessment Project survey Age: 7–12th grade Gender: 51% females Ethnicity: N/A Sexual orientation: N/A	Study design: quantitative, cross-sectional, secondary data analysis Setting: urban and suburban; 10 school districts in New England, USA Year of data collection: 2000–2001	Dating violence victimisation: Physical Sexual IPV CDC Youth Risk Behaviour Survey: yes/no to ‘beaten, pushed, slapped, injured with a weapon’ and ‘forced into sexual acts or raped’ on scale 0–6 of how many times	Impact of IPV on education: Grades (self-reported GPA) Perception of school environment = school attachment (fairness, enjoyment, quality and likelihood of dropping out)	Decrease in mental health Substance abuse	Direct: Decline in school attachment Indirect: Grade decline and drop out mediated through mental health illness and substance abuse	Limitations: Cross-sectional design, heterogeneous sample Future research: examine intersectionality (gender, social support, risk and protective factors); severity of violence; wider range of outcome measures; intervention/prevention research (i.e. real world implications)
Brewer et al. (2018)	Analysed sample: 85,071 random sample ACHA = American College Health Association data of NCHA = National College Health Assessment II b standardised survey Age: 18–24 Gender: 67.2% females Ethnicity: 65.2% Caucasian Sexual orientation: 90% heterosexual	Study design: Quantitative, cross-sectional, secondary data analysis Settings: urban and suburban; included USA regions (38% North East, 12% Midwest, 30% South and 20% West) Year of data collection: 2011–2014	Intimate partner violence abuse: Physical Sexual Psychological	Association between IPV and academic performance: Grades (self-reported cumulative GPA) Difficulties (yes/no answer)	Decline in mental health Psychological distress leads to poor physical health Suicidal thoughts and attempts	Indirect: Low GPA, decline in *academic performance* (experiencing difficulties) mediated through psychological distress	Limitations: Measurement of academic difficulties, larger time frame (i.e. more than last 12 months), low generalisability (small LGBQ sample) Future research: LGBTQ research (esp. bisexual), wider range of outcome measures, measurement of coercive control and different types of IPV
Chronister et al. (2014)	Analysed sample: Purposive 19 females Age: 13–18 Gender: Females only Ethnicity: 13 Caucasian, 3 Latinas, 1 Asian, 1 Jewish and 1 Native American Sexual orientation: N/A	Study design: Qualitative, cross-sectional; 1- to 1.5-h semi-structured face-to-face interviews Study’s 15-question guide around the impact of IPV on education, self-image and relationships Settings: Suburban and rural communities located in the Pacific Northwest, USA Year of data collection: Not mentioned	Unhealthy dating relationships: Physical (punching and slamming against the wall) Sexual (incl. coercion and being forced into sexual activities) Psychological (threats and ‘watching’)	Impact of IPV on educational experiences: Grades (failing) Absenteeism (lateness and truancy) Aspirations (graduating high school, attending college and motivation) Performance (loss of concentration and incomplete homework)	Mental health decline (depression, anxiety, self-harm, anger and mood swings, loss of positive self-image and self-worth, and trust issues) Physical health decline Social impact (isolation, loss of job and peer network) Substance abuse (marijuana, alcohol and hard drug abuse)	Direct: Decline in academic performance (incomplete homework and truancy due to sabotage/physical restraint), grades (missing classes and assignments), school transfer (expulsion and distance from abusers), and school engagement and motivation (sabotage and restraint) Indirect: Inability to concentrate mediated through depression/mental health decline; future orientation (change in vocational goals due to verbal abuse or coercion)	Limitations: Cross-sectional study, homogenous sample and self-reports Future research: intersection of cultural factors (race, ethnicity, gender and sexual orientation) and their influence on peer dynamics and abuse disclosure, more school-based studies and research on family intervention, and abusers’ interference with education and clarifying outcomes that are mediated or direct
Dank et al. (2014)	Analysed sample: 3745 young people in a relationship (85% response rate) Age: 7–12th grade Gender: 47.2% male, 52.3% female and 0.5% transgender Ethnicity: 73.7% White, 5% Black, 8.2% Latino/a, 2.2% Asian, 10.2% Mixed race and 0.7% Native American Sexual orientation: 93.8% heterosexual, 0.4 lesbian, 0.1 gay, 3.7 bisexual, 0.7 questioning, 0.3 queer and 1% other	Study design: Quantitative, cross-sectional; a large-scale paper–pencil survey Settings: urban, suburban and rural USA in New York, Pennsylvania and New Jersey Year of data collection: 2013	Dating abuse and coercion: Physical Sexual Psychological Cyber *Psychological* and *Physical Abuse Scales* (16 items on kicking, choking, beating, forced intercourse, insults, stalking and emotional manipulation) Control and Fear Scale (21 items around threats, monitoring or damaging property) *Cyberbullying Scale* (16 questions on pressure to send naked photos, threatening messages and using partners’ social media without permission)	Impact of IPV on school performance: Grades (self-reported, mostly AB/BC) Attendance (every week day or less)	Depressive symptoms (especially high for LGBT = transgender, homosexual and bisexual women) Anxiety Increase in aggression Involvement in delinquency and criminal activity	Direct: Decline in academic performance due to cyber abuse, ostracism/social isolation (threats from abusive partners) and truancy/lower attendance due to physical restraint Indirect: Lower attendance and grades also mediated through psychological distress, or maladjustment, and/or drug/alcohol abuse	Limitations: homogenous sample (middle school and White, potentially missed disadvantaged groups, truant students), self-reporting bias and cross-sectional study Future research: Direct vs. mediated outcomes; LGBTQ; intersection between race, gender identity, IPV and also socio-economic status

Edwards (2018)	Analysed sample: 25,122 high school students who answered New Hampshire Youth Risk Behaviour Survey by CDC Age: 13–18 Gender: 50.8% female and 49.2% male Ethnicity: 88% White; 5.5% Multiracial, 2.2% Black or African American, 1.4% American Indian or Alaskan Native and 1.9% Asian Sexual orientation: 90.5% heterosexual and 9.5% LGBQ	Study design: Quantitative, cross-sectional, secondary data analysis Settings: Non-specified (public high schools in the US) Year of data collection: 2013	Dating violence victimisation: Physical Sexual Survey yes and no answers to ‘beaten, pushed, slapped, injured with a weapon’ and ‘forced into sexual acts or intercourse, raped’ on scale 0–6 of how many times	Outcomes of IPV/academic performance: Grades (self-reported, mostly A/B…)	Depression, anxiety (especially high for LGBQ = homosexual, questioning and bisexual females) Binge drinking and substance abuse (particularly bisexual and questioning women/youth)	Direct: Low grades (truancy) Indirect: Low grades (mainly mediated by mental health issues for females)	Limitations: single-item measurements, self-reported grades (perception of poor performance varied – some saw C as poor performance and some did not) Future research: LGBQ youth and larger, more diverse samples; studies providing contextual information
Foshee et al. (2013)	Analysed sample: *N*= 3328 (response rate for each wave = 73–80%) Age: Wave I (8, 9 and 10 graders), Wave II and III six-month intervals, and Wave IV one year from Wave III (participants in 10, 11 and 12th grade) Gender: 49% male Ethnicity: 50% Black, 43% White, 7% other ethnicities (including Latin, mix race and Asian) Sexual orientation: Not mentioned	Study design: Quantitative, longitudinal (2.5 years), CDC-funded adolescent health-risk behaviours’ study Settings: 19 middle and high schools in two public school systems in predominantly rural US, North Carolina counties Years of data collection: 2003–2006	Dating abuse victimisation: Physical (including sexual) Psychological *Safe Dates Victimization Scales* with nine question investigating sexual and physical violence such as ‘how many times were you slapped, kicked, forced to have sex’ and four items listing psychological abuse such as being insulted or having to describe every minute of the day	Academic consequences of dating abuse: Grades (self-reported GPA) Aspirations (finishing high school and attending college)	Substance abuse among females Depression and anxiety Suicidal ideation	Direct/indirect: Neither type of violence predicted low academic aspirations or grades	Limitations: Retrospective bias or misreporting, low generalisability for rural sample, lack of academic performance measurements and vague definitions ‘going to a mall’ as the intent of engaging in a romantic relationship Future research: Studies focusing on cognitive, social and behavioural factors; also intersection of gender, support network; measuring consequences that vary by IPV type; more rural and urban/suburban comparative teen studies
Martz et al. (2016)	Analysed sample: *N*= 1003, convenience sample from Centre for Disease Control and Prevention’s Youth Risk Behavioural Surveillance study: Appalachian community Age: 9–12th grade Gender: 52% females and 48% males Ethnicity: 92.8% Caucasian and 7.2% non-White Sexual orientation: Not mentioned	Study design: Quantitative, cross-sectional, secondary data analysis CDC’s *Youth Risk Behaviour Survey* Settings: rural county school districts in western North Carolina, USA Year of data collection: 2011–2012	Dating violence victimisation: Physical Sexual Survey yes and no answers to ‘beaten, pushed, slapped, injured with a weapon’ and ‘forced into sexual acts or intercourse, raped’ on scale 0–6 of how many times	Association between dating violence and academic success: Grades (self-reported, mostly A/B…)	Depression and suicidal ideation Suicidal attempts Substance abuse (sexual violence only) Promiscuity and unprotected coitus	Direct: Lower probability of attending college Indirect: Low grades mediated through psychological distress	Limitations: Cross-sectional, only one item per victimisation type and grades – little outcomes measurement Future research: More studies on IPV among rural youth, include context: background of participants (i.e. sexual orientation and socio-economic status), SES, S.O. and gender-specific interventions
Strenio (2017)	Analysed sample: 4565 subpopulation National Longitudinal Study of Adolescent to Adult Health Age: 11–21 at T1 Gender: Male and female (55%) Ethnicity: Collected but % or number not indicated Sexual orientation: N/A	Study design: Quantitative, longitudinal survey Settings: urban and rural stratified sample in schools in the USA Years of data collection: 1994/5–2008/9	Intimate partner violence: Physical abuse Sexual abuse The Revised Conflict Tactics Scale (CTS) to measure physical and sexual violence victimisation	Association between IPV, severity and chronicity, with economic outcomes and education: High school graduation College completion	Economic hardship (26% of women) Need of welfare assistance (28%) Decreased earnings in adulthood	Decrease in high school graduation Unlikely enrolment into college Decrease in college completion	Limitations: Self-reported socio-economic status, no previous history of abuse/control for a variety and potential attrition of esp. vulnerable participants. Future research: More educational outcome measures and establishing causality with mediating factors such as SES or hardship.
Wiklund et al. (2010)	Analysed sample: Convenient purposive subsample of 2 women (initial 40 interviews with girls and young women 16–25, who sought help at a youth health centre for self-defined stress-related problems) Age: 18, 21 Gender: Females Ethnicity: Nationality Swedish Sexual orientation: Not mentioned	Study design: Qualitative, cross-sectional, exploratory open-ended interviews Participants asked about their health and stories, followed up with probing questions focused on ‘stress, health and lives’ (p. 209) Settings: City of Umeå, Sweden Year of data collection: 2005–2007	Interpersonal violence: Unhealthy relationships Themes emerged: Physical (locked up and beaten) Sexual (degradation and force) Psychological (name calling and coercion – guarded)	To illuminate the young women’s experiences of living in a violent relationship (education emerged as one of the themes) Themes emerged: Grades School attachment Attendance	Decline in mental health (anxiety, depression, trust issues, PTSD, worthlessness low self-esteem and suicidal thoughts) Physical health decline (weight loss, severe headaches and migraines, eating disorders, high blood pressure, fatigue and sleeplessness) Social consequences (quitting job and social isolation/loss of support network)	Direct: Truancy (physical restraint and forced to abuse substances), and year repetition and not continuing studies (sabotage) Indirect: Poor school attachment and low grades (mostly mediated through mental and physical issues)	Limitations: Not generalisable, cross-sectional, potential self-reporting bias, retrospective recall bias or researcher bias. Potential risk of misinterpretation, working with Swedish–English translations Future research: More studies observing long-term impact on adolescents' lives needed

## Discussion

This review was conducted in order to synthesise available literature as a means of increasing our understanding of how IPV impacts the educational well-being of young women. Furthermore, it aimed to critically evaluate the quality of the evidence and to identify gaps in research and literature. As such, our review of the literature was framed around two key research questions: What are the consequences of IPV on young women’s education? And what conclusions can be drawn from this literature review in terms of research gaps and limitations? As outlined earlier in the paper, the majority of articles reviewed point to a significant negative impact of IPV on young women’s education. It is important to note, however, the differences on how educational impact was conceptualised across the different studies as it ranged from negative impacts on grades to years of study completed, to delinquent behaviour, such as missing homework and truancy.

Nevertheless, across these different representations of educational outcomes, IPV was demonstrated as having a negative impact. Seven of the 10 studies reported a noticeable decline in grades being achieved, whilst others highlighted an increased detachment from school and academic progression and higher levels of truancy. One study has suggested that IPV reduced the likelihood of graduating high school, which increased with more severe levels of violence, and the probability of college graduation (Strenio, 2017). This is consistent with previous literature that suggests a correlation between abuse and trauma, and educational underachievement (Diette et al., 2017; Kennedy and Bennett, 2006). Indeed, previous ACE (adverse child experiences) research indicates negative impact on academic performance in children and young people (Karatekin & Ahluwalia, 2020; Symes et al., 2020).

Only one of the studies, that by Foshee et al. (2013), concluded that there was no long-term educational impact. A number of significant limitations need to be considered in relation to these findings, however. The authors themselves have highlighted that they did not consider ‘other aspects of an adolescent’s academic environment’ and by way of an example suggest that ‘school attendance could be negatively affected by the abuse’ (p. 8). Questions must also be raised about the instruments used to measure academic aspirations which centred on a vague query about ‘how important it was to: 1) graduate from high school, and 2) go to college’ (p. 5). The framing of this question left it somewhat impersonal, and respondents could easily have answered broadly rather than applying it to their own context. Additionally, in relation to grades achieved, students self-reported their most recent results in four subjects, which leaves open the possibility of inaccurate reporting and fails to explore longer-term performances prior to the commencement of the research. Indeed, this self-reporting of grades was a feature of several other studies (Banyard and Cross, 2008; Brewer et al., 2018).

Most importantly, a majority of the studies in the review were cross-sectional and half were based on secondary data sets, which meant that contextual and situational factors are largely unknown. The major drawback of this missing context is the uncertainty of whether an identified negative outcome, such as failing grades, for example, is actually a direct consequence of IPV or whether there is a mediating factor, such as substance abuse or mental health illness as a correlate or predictor of IPV as some of the studies suggest (for instance, Brewer et al., 2018; Foshee et al., 2013; Wiklund et al., 2010). This has major implications for our understanding fully the impact of IPV on education as it leaves open the possibility that other factors may be more significant in determining educational outcomes than the articles allow for. A further central element of this is better defining how educational outcomes are measured as discussed in the earlier paragraphs.

There are several other limitations to this review. For instance, the criteria included international research; however, only articles written in English were selected and nine studies out of the included 10 were conducted in the USA. Although the review was comprehensive, we only searched articles after 1981 in peer-reviewed journals which may have led to missing some relevant studies as well as grassroots-based research surrounding IPV or courtship violence. It is possible that some relevant papers were not included in the review due to missed terms associated with IPV; studies that included a sample older or younger than our pre-established age range and/or focused on multiple types of violence were also automatically excluded. The final count of female-only studies could be considered another limitation; the lack of research focused solely on young women meant that studies with male and female mixed samples had to be included in the final analysis.

Moreover, the findings presented in this review are based on only two qualitative studies with small samples and therefore, low generalisability; and although the remaining studies used data sets containing large samples, results based on eight studies present a major limitation to fully linking IPV with declining educational outputs. To strengthen conclusions, research needs to better contextualise and situate the data, and address threats to internal and external validity such as collecting data on educational performance prior to IPV and/or any mediating factors (i.e. mental health or substance abuse). It is also important to acknowledge that some studies relied on outdated data sets, some as old as 10 years. The fact that some studies estimate the prevalence of IPV among adolescents at 45% in both the UK and the USA (Fox et al., 2014; Gehring & Vaske, 2017) demonstrates it is an issue that needs increased attention in line with other, more established, social issues such as domestic violence and the traumatic legacy this exposes children and young people to (Evans et al., 2008).

## Conclusion

To date, 10 articles exist on IPV and its impact on education of young women aged 10–24. Future research should explore intimate abuse in educational settings, and based on the themes highlighted in this review, it would be particularly useful to give further attention to issues such as coercive control and cyber abuse. More longitudinal studies with heterogeneous samples, a wider range of outcomes and measures of victimisation, as well as those comparing rural and urban communities would also be beneficial in filling gaps in the research. Studies focusing on young women, LGBTQ+, and ethnic and racial minorities are especially needed, with specific attention paid to IPV and intersections of gender, socio-economic and/or immigration status, as well as ways in which social networks act as protective or risk factors (for an overview of implications, see Table 4).

**Table 4.** Implications of research, practice and policy.

Area	Implications
Research	More empirical research is needed on education and IPV/DV among young people with larger, more heterogeneous samples Studies with wider range of outcomes and measures of victimisation, longitudinal and intersectional studies (particularly with LGBTQ and ethnic/racial minorities), and comparative studies are needed (rural vs. urban)
Practice	Services accessed by survivors may benefit from context- and gender-specific interventions that consider individual factors such as sexual orientation, race/ethnicity, immigration or socio-economic status Recognising different types of violence that may have a different impact on young victims may also benefit practice and service users
Policy	Safeguarding policies at schools and governmental DV strategies/policies should focus on inclusion and diversity (i.e. risk factors and intersections of IPV) as well as cyber abuse and coercive control Mandatory training is needed at schools and other related professions that recognises the impact of IPV on education and how it manifests (such as disengagement or over-commitment)

Certainly, the findings point to the need for further research that could have important implications for policy approaches to the issue. As such, research should continue to analyse the short-term impacts of IPV among young people to assist in making it more identifiable and this research should focus on addressing some of the limitations of the studies highlighted in this review. A final aspect arising from the review is the much longer-term repercussions that can result from IPV without meaningful and effective interventions. Research has consistently demonstrated the social and individual benefits of education with Riddell highlighting how those ‘who acquire additional schooling generally earn more over their lifetimes, achieve higher levels of employment, and enjoy more satisfying careers’ (Riddell, 2005, p. 138). The literature examined in this review has highlighted the negative impact IPV has and how this has serious longer-term repercussions.
